# Species and Strain Glycosylation Patterns of PrP^Sc^

**DOI:** 10.1371/journal.pone.0005633

**Published:** 2009-05-20

**Authors:** Konstantinos Xanthopoulos, Magdalini Polymenidou, Sue J. Bellworthy, Sylvie L. Benestad, Theodoros Sklaviadis

**Affiliations:** 1 Laboratory of Pharmacology, Department of Pharmacy, Aristotle University of Thessaloniki, Thessaloniki, Greece; 2 Ludwig Institute for Cancer Research, and the Department of Cellular and Molecular Medicine, University of California San Diego, La Jolla, California, United States of America; 3 Pathology Department, Veterinary Laboratories Agency-Weybridge, Addlestone, Surrey, United Kingdom; 4 Department of Pathology, National Veterinary Institute, Oslo, Norway; 5 Institute of Agrobiotechnology, Centre for Research and Technology, Thermi, Greece; Massachusetts Institute of Technology, United States of America

## Abstract

**Background:**

A key event in transmissible spongiform encephalopathies (TSEs) is the conversion of the soluble, protease-sensitive glycosylated prion protein (PrP^C^) to an abnormally structured, aggregated and partially protease-resistant isoform (PrP^Sc^). Both PrP isoforms bear two potential glycosylation sites and thus in a typical western blot with an anti-PrP antibody three distinct bands appear, corresponding to the di-, mono- or unglycosylated forms of the protein. The relative intensity and electrophoretic mobility of the three bands are characteristic of each TSE strain and have been used to discriminate between them.

**Methodology/Principal Findings:**

In the present study we used lectin-based western blotting to evaluate possible variations in composition within sugar chains carried by PrP^Sc^ purified from subjects affected with different TSEs. Our findings indicate that in addition to the already well-documented differences in electrophoretic mobility and amounts of the glycosylated PrP^Sc^ forms, TSE strains also vary in the abundance of specific N-linked sugars of the PrP^Sc^ protein.

**Conclusions/Significance:**

These results imply that PrP glycosylation might fine-tune the conversion of PrP^C^ to PrP^Sc^ and could play an accessory role in the appearance of some of the characteristic features of TSE strains. The differences in sugar composition could also be used as an additional tool for discrimination between the various TSEs.

## Introduction

Transmissible Spongiform Encephalopathies (TSEs) are invariably fatal neurodegenerative diseases affecting both humans and animals. TSEs include sporadic and variant Creutzfeldt Jakob Disease (sCJD, vCJD) in humans, scrapie (Sc) in sheep and goats and bovine spongiform encephalopathy (BSE) in cattle. The common pathogen to all TSEs is the ‘prion’, the major component of which is an abnormal isoform (PrP^Sc^) of the cellular prion protein (PrP^C^). It is believed that upon prion infection, PrP^Sc^ elicits the conversion of PrP^C^ to the abnormal conformer [1]. The two isoforms share the same primary structure, but differ in the secondary; PrP^Sc^ consists mainly of β-pleated sheet [2], while PrP^C^ contains a two-stranded antiparallel beta-sheet and three alpha-helices [3], [4]. Unlike PrP^C^, PrP^Sc^ is partially detergent insoluble, protease- and heat-resistant [5] and a large proportion of its methionine residues is sulfoxided [6].

PrP consists of a single polypeptide chain which is attached to the cell surface by a glycosylphosphatidylinositol (GPI) anchor at its carboxyl terminus [7] and bears two highly conserved N-glycosylation sites [8]. Depending on the extent of glycosylation, the mature prion protein can be unglycosylated, mono- or diglycosylated. TSE strains display differences in abundance and electrophoretic mobility of the proteinase K (PK) resistant forms of PrP^Sc^ glycoforms and these differences have been used extensively as a method to discriminate between the various TSE strains, through a process known as glycotyping [9]–[11].

Glycosylation is a major contributor to prion protein's heterogeneity; numerous PrP^Sc^ subpopulations differing in N-linked glycans have been identified when PrP^Sc^ populations purified from hamster [12], [13] or murine [14] brains were analyzed. Similarly, heterogeneity in the GPI anchor composition has also been observed [15].

PrP^C^ and PrP^Sc^ contain different but overlapping sets of glycan structures. The proportion of these differs and an increase in tri- and tetra-antennary glycans in PrP^Sc^ has been reported [12]. TSEs also exhibit differences in PrP^Sc^ glycosylation patterns; Ricinus communis agglutinin I (RCA I) binding to PrP^Sc^ varies between vCJD- and sCJD-derived PrP^Sc^ [16] and ELISAs with RCA I and Datura stramonium lectin (DSL) can be used to discriminate between normal and scrapie affected tissues [17]. Differences in glycosylation patterns of PrP, however, are not necessarily associated with TSE pathogenesis, since the brain area from which PrP was purified [18], [19], aging [20] and cell differentiation state [21] have also been shown to affect glycosylation of the prion protein.

The effect of glycosylation on PrP^C^ function remains obscure. PrP^C^ N-glycosylation differences have been linked to maturation of progenitor 1C11 neuronal cells to serotonergic or nor-adrenergic neurons. These glycosylation differences may be linked to some neurospecific functions of PrP^C^, and the specific brain targeting of prion strains [21]. However the effect of N-linked glycosylation on prion strains and TSEs propagation is not yet fully understood. Initial reports indicated it is not required for the conversion of PrP^C^ to PrP^Sc^ [22]. More recent data from both cell culture systems and transgenic animals also argue that diglycosylation is dispensable for PrP^Sc^ formation [23], whereas in a different study, in which PMCA generated unglycosylated PrP^Sc^ was used as the infectious agent, it was shown that strain tropism, as well as other strain associated features, can be propagated in the absence of the glycans [24]. On the other hand, a series of reports suggest that N-linked glycosylation may modulate the conversion [25]–[30].

In this study, we aimed to systematically study glycosylation differences between PrP^Sc^ populations purified from various TSEs, by refining the glycotyping procedure. This was achieved by blotting the sugar moieties that form part of the PrP^Sc^ glycosyl chains in different TSEs with lectins, which enabled estimation of the glycans N-linked to the protein. Our analysis indicates that the abundance of specific subsets of N-linked sugars varies among various TSEs. This variation could be linked to specific PrP^Sc^ conformation required for efficient propagation by each TSE strain, as well as to some of the TSE strains features and could be used as an additional criterion in identification of TSE strain origin.

## Materials and Methods

### Tissues and reagents

Murine (301 V), ovine and bovine BSE samples were obtained from VLA, Weybridge, UK. Normal and scrapie ovine samples were a gift from Dr. P. Toumazos (Veterinary Sevices Laboratory, Nicosia, Cyprus). Nor98 ovine samples were provided by Dr S. Benestad (National Veterinary Institute, Oslo, Norway). Sporadic CJD brain samples were obtained from confirmed sCJD cases [31]. Murine scrapie (RML) was generated in our laboratory. All samples were stored at −80°C until use. Whole brains were processed in all murine cases. Tissue from the brain stem was used on all other occasions, unless otherwise indicated. The ovine samples used in the study were from sheep with ARQ/ARQ genotype. Handling of infectious material was carried out in a safety level 3 facility. Animal work has been conducted following relevant national and EU regulations.

Proteinase K and all chemicals were purchased from Sigma-Aldrich (St. Louis, MO, U.S.A.). Biotinylated lectins and ABC complex were acquired from Vector Laboratories (Burlingame, CA, U.S.A.). Two dimensional (2D) electrophoresis system (Zoom IPGrunner) and all reagents required for 2D-electrophoresis were purchased from Invitrogen (Carlsbad, CA, U.S.A.). Monoclonal anti-PrP antibody 6H4 was a generous gift from Prionics (Schlieren, Switzerland), while the anti-PrP antibody P4 [32] was a gift from Dr. M.H. Groschup (INEID, Greifswald-Insel Riems, Germany). Horseradish Peroxidase (HRP) and Alkaline Phosphatase (AP) conjugated Rabbit anti-Mouse IgG, Enhanced Chemiluminescence (ECL) and SuperSignal West Femto Maximum Sensitivity western blotting substrates were purchased from Pierce (Rockford, IL, U.S.A.). Prestained molecular mass markers, CDP-star western blotting substrate and the Peptide: N-Glycosidase F (PNGase F) kit were from NEB (Ipswich, MA, U.S.A.).

#### PrP^Sc^ purification with the guanidinium protocol

The short purification protocol proposed by Polymenidou and colleagues [33] was modified to allow purification of PrP^Sc^ from small amounts of tissue. Ten percent *w/v* homogenates were prepared in homogenization buffer [0.01 M Tris-HCl, 0.15 M NaCl, 5 mM EDTA (pH 8.0), 1% *v/v* Triton X-100, 0.5% *w/v* sodium deoxycholate], using a FastPrep homogenizer (Thermo Scientific, Waltham, MA, U.S.A.). 200 µl of the homogenates were thoroughly mixed with 100 µl of 1 M Guanidinium HCl and incubated at 25°C for 1 h with constant agitation. The samples were then centrifuged for 20 min at 21,000×*g* over 25 µl of 20% *w/v* sucrose in homogenization buffer. Following centrifugation, the pellet was resuspended in 200 µl of homogenization buffer and treated with PK for 1 h at 37°C (murine samples: 30 µg/ml; ovine and bovine samples: 75 µg/ml). The reaction was stopped with the addition of PMSF to a final concentration of 5 mM and then 300 µl of cold phosphate buffered saline (PBS) and 500 µl of cold precipitation buffer (20% *w/v* NaCl, 0.1% *w/v* N-lauroyl sarcosine in PBS) were added. The samples were incubated for 10 min at −20°C and then centrifuged at 24,000×*g* for 15 min. The supernatant was removed and the pellet was washed with 25 mM Tris-HCl buffer (pH 8.8), 0.05% *w/v* N-lauroyl sarcosine, before being centrifuged again at 24,000×*g* for 10 min. The supernatant was discarded and the pellet was left overnight at −80°C in absolute MeOH. The sample was centrifuged again for 30 min at 24,000×*g*, the supernatant was discarded and the pellet was resuspended in 2.5× O' Farrell sample loading buffer.

#### SDS-PAGE and electrotransfer

The samples were incubated at 100°C for 10 min and then centrifuged for 2 min at 24,000×*g*. The proteins in the samples were separated by SDS-PAGE on 13% *w/v* polyacrylamide gels and then transferred to Polyvinylidene Fluoride (PVDF) membranes (Imobillon, Billerica, MA, U.S.A.) using a mini-transblot cell for 2.5 h at 100 V.

#### Two dimensional (2D) electrophoresis

2D electrophoresis was performed using the Invitrogen IPGrunner system. PrP^Sc^ was purified with the guanidinium protocol and the final pellet was resuspended in rehydration buffer [6 M urea, 2 M thiourea, 4% *v/v* Zoom Carrier Ampholytes (pH 3–10), 2% *w/v* CHAPS and 60 mM dithiothreitol (DTT)]. Isoelectric focusing was performed with 7 cm long, pH 3–10 linear immobilized pH gradient strips. Each strip was rehydrated overnight at 25°C in 200 µl rehydration buffer containing PrP^Sc^ purified from 50 mg tissue and then electrophoresed (200 V for 20 min, 450 V for 15 min, 750 V for 15 min, 2000 V for 90 min). Prior to running the second dimension, the strips were equilibrated for 10 min in 1× NuPAGE LDS sample buffer supplemented with 6 M urea, 2 M thiourea, 60 mM DTT and then for 10 min in 1× NuPAGE LDS sample buffer containing 6 M urea, 2 M thiourea and 2.5% *w/v* iodoacetamide. The focused proteins were separated on a 4–12% NuPAGE bis-tris gel, using MES buffer. Following electrophoresis the proteins were electrotransferred on PVDF membranes as previously outlined.

#### Antibody blotting

For antibody blotting, the PVDF membrane was blocked for 1 h at room temperature with blocking buffer [5% *w/v* non-fat dry milk in PBS containing 0.1% *v/v* Tween 20 (PBST)] and incubated overnight at 4°C with the primary antibody diluted in blocking buffer (6H4: 0.2 µg/ml; P4: 1∶500 *v/v*). After washing with PBST, the membrane was incubated for 1 h at room temperature with HRP- or AP-conjugated rabbit anti-mouse IgG (depending on the development method) at a concentration of 0.1 µg/ml in blocking buffer. Immunoreactivity was visualized on x-ray films by ECL, West Femto or CDP-star, as specified by the manufacturer.

#### Lectin blotting

Following electrotransfer, the membrane was blocked with PBST, incubated for 1 h at room temperature with the biotinylated lectin diluted in PBST (DSL: 6 µg/ml; RCA I and other lectins: 10 µg/ml) and washed with PBST. The membrane was then incubated with HRP-conjugated ABC complex for 35 min at room temperature and washed with PBST. Lectin binding to PrP^Sc^ was visualized on x-ray films using ECL or West Femto substrate, following manufacturer's directions.

On some occasions the same membrane was blotted with both the biotinylated lectin and the antibody. In that case, the membrane was first probed with the lectin and afterwards with the antibody, following the aforementioned protocols. To avoid cross-reactivity between the two procedures, the membrane was incubated for at least 48 h in PBST prior to antibody incubation and loss of previous signals was verified by long exposures of the membrane to x-ray films. To visualize the bound antibody, AP-conjugated secondary antibody and CDP-star reagent were used.

#### Densitometric analysis

Blots were scanned and the intensities of the bands that corresponded to the di- and the monoglycosylated forms of PrP^Sc^ were estimated using ImageJ software (v 1.40 g, available at http://rsbweb.nih.gov/ij/download.html). For densitometric analysis only exposures within the linear range of the x-ray film were used. To facilitate comparisons, the IOD_l_/IOD_ab_ ratio (IOD_l_: integrated optical density of the lectin reactive band; IOD_ab_: integrated optical density of the antibody reactive band) was calculated for both lectins and both glycosylated forms of PrP^Sc^. Unless otherwise indicated, the IOD_l_ and the IOD_ab_ were calculated from x-ray films developed with identical methods and exposed for equal times on the blots. The IOD_l_/IOD_ab_ ratio was used as an indicator of the abundance of PrP^Sc^ molecules which carry ‘suitable’ glycan moieties that permit specific lectin binding within the total PrP^Sc^ population, which is recognized by the antibody. Differences in the IOD_l_/IOD_ab_ ratios between different TSEs were tested for statistical significance with the Mann Whitney U test. All statistical tests were performed with data from three individuals per group. The procedures described were performed at least thrice to ensure reproducibility of results.

## Results

To minimize the risk of non-specific binding, initial screenings for lectin binding were performed on scrapie associated fibrils (SAFs) preparations, which contain highly purified PrP^Sc^. A number of lectins recognizing N-linked glycans, including Solanum tuberosum lectin (STL), which recognizes oligomers of N-acetylglucosamine, Wheat germ agglutinin (WGA), which preferentially recognizes dimers and trimers of N-acetylglucosamine, and Maackia amurensis lectin II (MAL II), which recognizes sialic acid in an (α→2,3) linkage, either failed to produce any signal (STL, MAL II) or produced a very faint one (WGA recognized the di-glycosylated form). Others, including Concanavalin A, which recognizes α-linked mannose, did not recognize PrP^Sc^ and produced high non-specific binding. Results from these initial screenings are summarized in Supplementary Table S1. Of the positively reacting lectins, the ones producing the most specific signal were Datura stramonium agglutinin (DSL), which recognizes (β→1,4) linked N-acetylglucosamine oligomers and Ricinus communis agglutinin I (RCA I), which recognizes oligosaccharides ending in galactose and N-acetylgalactosamine (Fig S1 and data not shown).

We next used DSL and RCA I to probe guanidinium protocol-purified PrP^Sc^. The guanidinium protocol was devised due to the limited tissue availability, which prohibited the use of SAF preparations. Lectin specificity for PrP^Sc^ was confirmed by applying the guanidinium purification and blotting protocols on simultaneously processed normal and TSE-affected tissue. No proteins were probed by the antibody or the lectins in the normal sample, as opposed to the TSE affected sample, in which the lectins recognized di- and monoglycosylated PrP^Sc^ (Fig S2C).

### 

#### 2D electrophoresis

**Figure 1:**
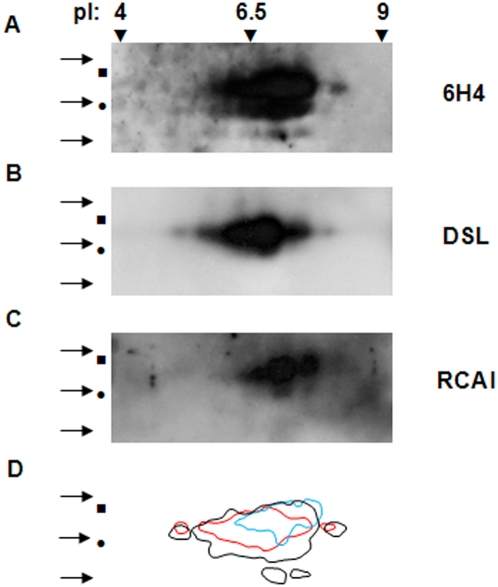
Two dimensional electrophoresis and lectin blotting of PrP^Sc^ purified with the guanidinium protocol. PrP^Sc^ was purified from a representative ovine scrapie sample with the guanidinium protocol, resolved in 2D and blotted with 6H4 (A), DSL (B), and RCA I (C), as outlined in ‘Materials and Methods’. Squares (▪): diglycosylated PrP^Sc^; bullets (•): monoglycosylated; arrows (→): 32, 25, 16.5 kDa molecular mass markers. (D) Superimposition of the cluster of DSL- (red line) and RCA I- (blue line) reactive spots on the cluster of 6H4-immunoreactive spots (black line).

To further validate lectin specificity for PrP^Sc^ in guanidinium protocol treated preparations we performed 2D electrophoresis. The enhanced resolution afforded by this method minimizes the probability of bands overlapping, which could occur in one dimensional electrophoresis and lead to false positive results. PrP^Sc^ purified with the guanidinium protocol from a representative ovine scrapie preparation was analyzed by 2D electrophoresis and then probed with the monoclonal antibody 6H4 and the lectins. Upon blotting with 6H4 a cluster of immunoreactive spots covering a range of apparent molecular masses corresponding to the PK treated di-, mono- and unglycosylated forms of PrP^Sc^ was recognized. The isoelectric points (pIs) of this cluster of spots ranged from ∼5 to 8 (Fig 1A). When identically prepared membranes were blotted with the two lectins, a cluster of spots with apparent molecular masses corresponding to the di- and the monoglycosylated forms of PK treated PrP^Sc^ was obtained with both lectins. The DSL-reacting cluster of spots was smaller in area than the 6H4-reacting one, but had an almost similar pI range (∼5–8) distribution (Fig 1A, B, D). RCA I, recognized a smaller cluster of spots than both DSL and 6H4. The pIs of the spots in this cluster ranged between ∼6.5 and 8. (Fig 1A, C, D). The resolution of the 2Ds was not high enough to allow for individual spots to be visualized, however 2D blotting with the antibody and the lectins showed that the lectin-reacting proteins are a subpopulation of the antibody-reacting ones, as evidenced in Fig 1D. Thus 2D blotting proved that DSL and RCA I specifically blot PrP^Sc^ and that these lectins recognize a subset only of 6H4 reacting PrP^Sc^ molecules.

#### Ovine scrapie and bovine BSE preparations

**Figure 2:**
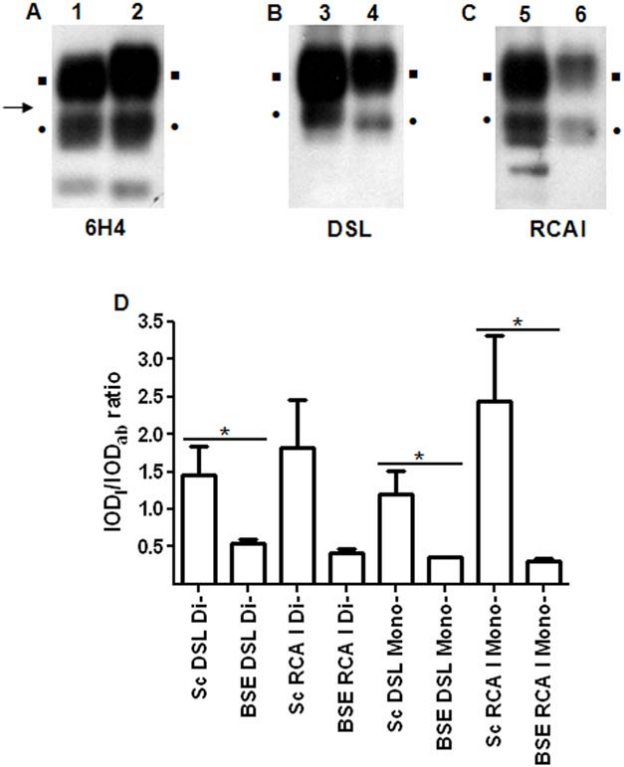
Ovine scrapie and bovine BSE. PrP^Sc^ was purified from ovine scrapie (Lanes 1, 3, 5) and bovine BSE (Lanes 2, 4, 6) samples with the guanidinium protocol and probed with 6H4 (A), DSL (B), and RCA I (C), as outlined in ‘Materials and Methods’. Equal amounts of starting material were loaded in all lanes and equal exposure times were used for all three panels. Squares (▪): diglycosylated PrP^Sc^; bullets (•): monoglycosylated; arrow (→): 25 kDa molecular mass marker. The remaining non-marked lectin reactive glycoproteins should be considered as interfering glycoproteins. (D) Densitometric analysis of the diglycosylated and the monoglycosylated forms of PrP^Sc^. IOD_l_/IOD_ab_ is the ratio of the IOD of the lectin- blotted band versus the IOD of the antibody-blotted band, estimated as described in ‘Materials and Methods’. Columns represent the mean IOD_l_/IOD_ab_ and error bars the SEM from three individuals. *P<0.05.

We next applied the method on a variety of TSE affected samples. When ovine scrapie (Fig 2; Lanes 1, 3, 5) and bovine BSE PrP^Sc^ (Fig 2; Lanes 2, 4, 6) were purified and probed with 6H4, the three-banded pattern was obtained. As expected, unglycosylated PrP^Sc^ migrated faster in the bovine BSE sample (Fig 2A). Upon lectin blotting, the lectins recognized di- and monoglycosylated PrP^Sc^ in both bovine BSE and ovine scrapie samples (Fig 2B–C). Densitometric analysis was performed as described in ‘Materials and Methods’ and the IOD_l_/IOD_ab_ ratio was found on all occasions to be lower in the bovine BSE samples (Fig 2D). These differences were more pronounced in the RCA I blotted monoglycosylated PrP^Sc^ bands; the IOD_l_/IOD_ab_ of the ovine scrapie samples was ∼8-fold greater than the IOD_l_/IOD_ab_ of the bovine BSE samples. Significantly different (P<0.05) IOD_l_/IOD_ab_ ratios were obtained on all comparisons, except for RCA I blotting of diglycosylated PrP^Sc^.

In addition to differences in the intensities of the bands, small differences in the apparent molecular mass of monoglycosylated PrP^Sc^ were noticed following antibody and DSL blotting. With antibody blotting, the monoglycosylated PrP^Sc^ band was rather diffuse and displayed the same apparent molecular mass in both ovine scrapie and bovine BSE samples. Following DSL blotting, monoglycosylated PrP^Sc^ was sharper in both ovine scrapie and bovine BSE samples and the lectin appeared to have a preference for different subpopulations of 6H4-reactive monoglycosylated PrP^Sc^; DSL recognized the higher apparent molecular mass region of the 6H4-reacting monoglycosylated PrP^Sc^ band in ovine scrapie samples, whereas in bovine BSE samples it blotted the medium apparent molecular mass region of the 6H4-reacting monoglycosylated PrP^Sc^ band. To better exhibit this preference for the higher molecular mass subpopulation of monoglycosylated PrP^Sc^ in ovine scrapie samples, guanidinium protocol purified PrP^Sc^ from two representative ovine scrapie samples was electrophoresed and electrotransferred. Following electrotransfer, the membrane was cut and one part blotted with 6H4 and the other with DSL. Meticulous alignment of the two parts revealed the differences in apparent molecular weight of monoglycosylated PrP^Sc^ following antibody or lectin staining (Fig S3).

#### Ovine scrapie and BSE preparations

**Figure 3:**
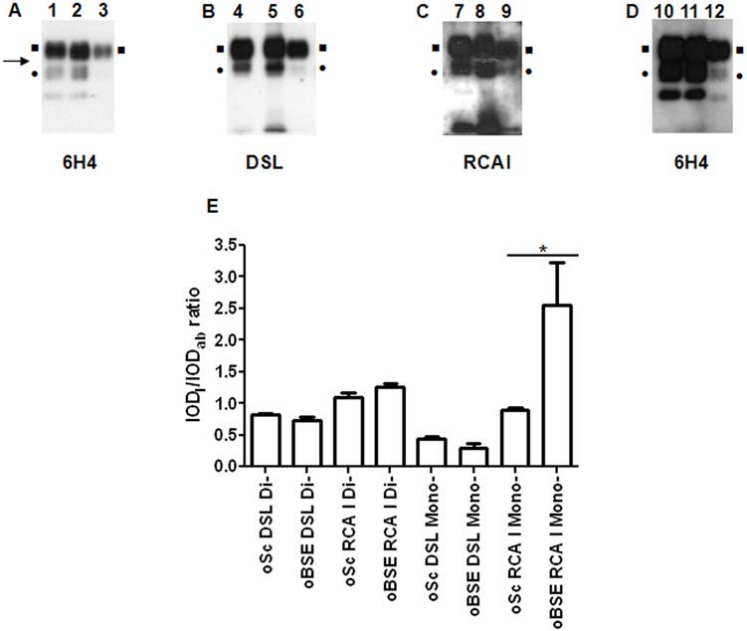
Ovine BSE. 6H4 (A), DSL (B), and RCA I (C) blotting of PrP^Sc^ purified from ovine scrapie (Lanes 1, 2, 4, 5, 7, 8, 10, 11) and ovine BSE (Lanes 3, 6, 9, 12) with the guanidinium protocol and developed with ECL, as described in ‘Materials and Methods’. Equal amounts of starting material were loaded in all lanes and equal exposure times were used for all three panels. (D) Development of 6H4 blotted lanes with a higher sensitivity substrate (West Femto, Lanes 10, 11, 12). Squares (▪): diglycosylated PrP^Sc^; bullets (•): monoglycosylated; arrow (→): 25 kDa molecular mass marker. The remaining non-marked lectin-reactive glycoproteins should be considered as interfering glycoproteins. (E) Densitometric analysis of the diglycosylated and the monoglycosylated forms of PrP^Sc^. The IOD_l_/IOD_ab_ ratio was calculated as outlined in ‘Materials and Methods’, but the IOD_ab_ following development with the high sensitivity substrate and thus a different exposure time was used. Columns represent the mean IOD_l_/IOD_ab_ and error bars the SEM from three individuals. *P<0.05.

In guanidinium protocol preparations of ovine BSE samples virtually no signal was obtained for the mono- and the unglycosylated forms of PrP^Sc^ upon antibody (6H4) blotting using standard sensitivity substrate (ECL, Fig 3A; Lane 3). On the contrary, equal loads of parallel-processed ovine scrapie samples produced the typical three-banded pattern (Fig 3A; Lanes 1, 2). To visualize the antibody reactive mono- and unglycosylated PrP^Sc^ bands in ovine BSE preparations, the blots had to be developed with a higher sensitivity substrate (SuperSignal West Femto, Fig 3D). As opposed to antibody blotting, PrP^Sc^ from ovine BSE samples was recognized by the lectins and a clear signal was obtained even with ECL (Fig 3; Lanes 3, 6, 9).

Due to the extremely faint ECL-developed, antibody-blotted monoglycosylated PrP^Sc^ band in the ovine BSE samples, densitometry data from the West Femto-developed antibody blots was used to calculate the IOD_l_/IOD_ab_ ratio for all samples in the densitometric analysis (Fig 3E). The analysis revealed a statistically significant (P<0.05) difference in the intensity of the RCA I reactive monoglycosylated band between the BSE and scrapie challenged ovine samples.

Similarly to the results obtained in ovine scrapie and bovine BSE samples, DSL-blotted, ovine BSE monoglycosylated PrP^Sc^ displayed lower apparent molecular mass than ovine scrapie monoglycosylated PrP^Sc^ (Fig 3B).

#### Murine scrapie and BSE preparations

**Figure 4:**
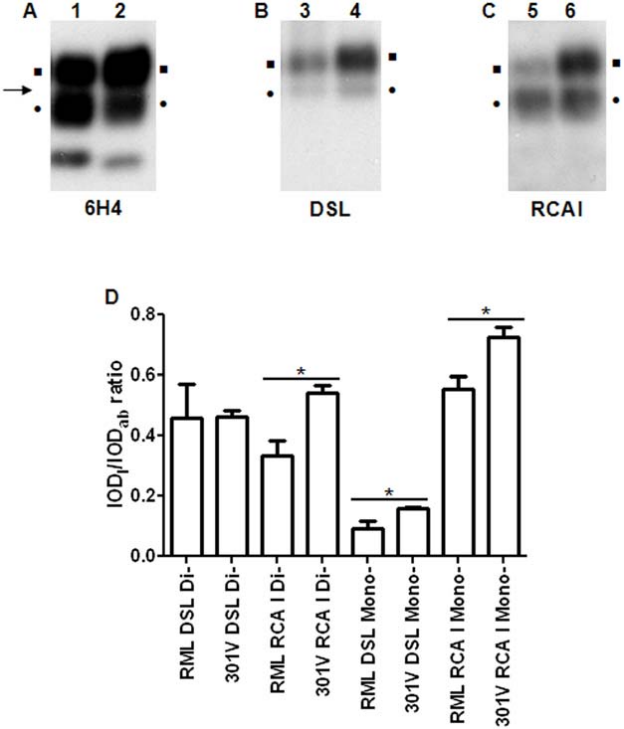
Murine scrapie and BSE. 6H4 (A), DSL (B) and RCA I (C) blotting of PrP^Sc^ purified from murine scrapie (Lanes 1, 3, 5) and murine BSE (Lanes 2, 4, 6) samples, following the guanidinium protocol. Equal amounts of starting material were loaded in all lanes and equal exposure times were used for all three panels. Squares (▪): diglycosylated PrP^Sc^; bullets (•): monoglycosylated; arrow (→): 25 kDa molecular mass marker. (D) Densitometric analysis of the diglycosylated and the monoglycosylated forms of PrP^Sc^. The intensity of the band of the di- and the monoglycosylated PrP^Sc^ was estimated and the IOD_l_/IOD_ab_ ratio was calculated, as outlined in ‘Materials and Methods’. Columns represent the mean IOD_l_/IOD_ab_ and error bars the SEM from three individuals. *P<0.05.

Similar analyses were performed on murine scrapie (RML) and BSE (301 V) samples (Fig 4; Lanes 1, 3, 5 and 2, 4, 6 respectively). The blots were probed with 6H4 and the three-banded pattern for PrP^Sc^ was obtained (Fig 4A) on all samples. Monoglycosylated PrP^Sc^ in murine BSE samples migrated slightly faster than its counterpart in murine scrapie samples. Upon lectin blotting of the same preparations, very faint blotting was produced in murine scrapie, as opposed to murine BSE samples (Fig 4B, C; Lanes 3–6). These differences in blotting were evident in the densitometric analysis, where statistically significant differences emerged on most comparisons (Fig 4D). Faint staining of murine scrapie samples hampered thorough analysis of differences in the apparent molecular masses of the lectin-blotted bands. However, lectin blotted bands were sharper than the corresponding antibody blotted ones, implying a preference of the lectins for specific subsets of PrP^Sc^ molecules (Fig 4 A, B).

## Discussion

In this study, we intended to determine whether differences occur in the composition of N-linked sugar chains of PrP^Sc^ populations derived from different TSEs. To address this question, we blotted PrP^Sc^ purified from tissue affected with different TSEs with monoclonal antibodies and lectins. Lectins are carbohydrate-binding proteins, which recognize and bind to specific sugar moieties with high specificity [34].

We initially screened a panel of lectins for their ability to recognize PrP^Sc^ in western blots. From this screening two lectins with affinity for PrP^Sc^ emerged, namely DSL and RCA I. DSL recognizes repeating N-acetyllactosamine [Gal β(1→4) GlcNAc] oligomers, whereas RCA I binds to terminal galactose (Gal) or N-acetylgalactosamine (GalNAc). Since PrP^Sc^ from naturally occurring TSEs contains complex-type sugars composed of multiple units of lactosamines, which are multimers of Gal β(1→4) GlcNAc [12]–[14], DSL and RCA I were expected to bind PrP^Sc^. In agreement with that, DSL and RCA I were previously reported to bind PrP in ELISAs [16], [17]. Our initial findings were confirmed by a series of results, including: i) blotting of PrP^Sc^ from highly purified PrP^Sc^ preparations (SAFs) from a multitude of species with the lectins, (Fig S1), ii) lack of lectin blotting in PK treated preparations from normal animals (Fig S2C), and in PNGase and PK treated preparations of TSE affected animals (data not shown), iii) colocalization of lectins and antibody (6H4) reactivity following 2D electrophoresis of purified PrP^Sc^ (Fig 1). These data further confirm that DSL and RCA I recognize specifically PrP^Sc^ subpopulations and not other copurifying host glycoproteins.

Once the protocols for PrP^Sc^ purification and lectin blotting were devised, we studied the glycosylation differences in PrP^Sc^ populations purified from tissues affected with various TSEs. We found that DSL and RCA I blotted PrP^Sc^ in all samples. This result indicates that the purified PrP^Sc^ populations contain similar glycan species. However, blotting intensity of the glycosylated bands varied, suggesting that the abundance of each particular type of glycan in PrP^Sc^ populations differs among various TSEs.

Lectin blotting following PrP^Sc^ purification with the guanidinium protocol was also used to study glycosylation differences between classical and atypical (Nor98) scrapie cases (Fig S4). Results from lectin blotting of Nor98 scrapie PrP^Sc^ were difficult to interpret, but appear to be in agreement with recently published data [35], [36] and although non conclusive, demonstrate that PrP^Sc^ glycosylation pattern is different than classical scrapie. The appearance of a series of glycosylated PrP^Sc^ fragments with apparent molecular masses ranging from 21 to 31 kDa in PK treated Nor98 samples has already been suggested. These are proposed to arise from the glycosylation of PrP^Sc^ fragments with molecular masses of 18 and 23 kDa [35]. In our study, lectin reactivity was observed in this range of molecular masses and possibly some of the observed bands could correspond to these Nor98 PrP^Sc^ fragments.

A P4 reactive, glycosylated and PK resistant PrP^Sc^ fragment, with an apparent molecular mass of 33 kDa has already been described in Nor98 samples [36]. This fragment was most probably recognized by both DSL and RCA I, as evidenced by the P4 and lectins reactivity of a 33 kDa band (Fig S4B, D open diamond). Another glycosylated and PK resistant PrP^Sc^ fragment with an apparent molecular mass of 24 kDa, which bears the L42, but not the P4 epitope, has also been described in Nor98 samples [36]. Since the L42 antibody was not used in our study, positive identification of this fragment among the lectin reacting bands was difficult. However both lectins and mostly DSL recognize a band with an apparent electrophoretic mobility of approximately 22 kDa, which could correspond to the 24 kDa PrP^Sc^ fragment (Fig S4B, D solid diamond). Interestingly, both lectins and especially RCA I clearly recognized two bands with molecular masses ranging between 6 and 8 kDa (Fig S4B, D, open squares). These two bands may correspond to C-terminal PrP^Sc^ fragments encompassing the two glycosylation sites, but not the P4 epitope.

It is important to stress that due to limitations of our experimental approach, we cannot rule out the possibility that any of the bands that were associated with PrP^Sc^ in Nor98 samples are in fact interfering glycoproteins with an electrophoretic mobility similar to some PrP^Sc^ fragments. These results await confirmation by mass spectrometry and/or amino acid sequencing, which due to limited availability of tissue have not yet been performed.

PrP^Sc^ which is purified from different species is heterogeneous with regard to sugar content [12]–[14]; each subpopulation of PrP^Sc^ molecules contains different N-linked sugars, and thus may or may not be recognized by the lectins in use. This recognition is only possible if the PrP^Sc^ molecule carries the ‘suitable’ sugar moiety for the lectin to bind. This was evidenced in our results by the differences in signal intensity and electrophoretic mobility of the bands that were recognized by the antibody and the lectins. Lectins recognize a subpopulation only within the total PrP^Sc^ population. The strong signal that was obtained for monoglycosylated PrP^Sc^ in ovine BSE samples following lectin-blotting, indicates that this preparation is enriched in a lectin-reacting PrP^Sc^ subpopulation, leading to strong reactivity with the lectins, in spite of the poor antibody reactivity. Similarly, the differences in apparent molecular mass of antibody- and lectin-reactive bands (Fig 2, 3 and S3) should be attributed to the fact that only some of the purified PrP^Sc^ molecules are blotted by the lectins. As a result, DSL binds only part of the monoglycosylated PrP^Sc^ molecules and the corresponding band appears sharper than the antibody blotted one. In agreement with these observations, part of the 6H4 immunoreactive cluster of spots is also recognized by the lectins in 2D blots (Fig 1).

It is important to point out that different PrP^Sc^ purification protocols may be biased towards different PrP^Sc^ subpopulations and possibly permit co-purification of interfering glycoproteins. Four different purification protocols were evaluated in our study: the short purification process [33], the sodium phosphotungstate protocol (NaPTA) [37], the SAF preparations [38] and the guanidinium protocol. Although all four purification protocols purified PrP^Sc^ with comparable glycoform ratios (Fig S2D), the presence of interfering glycoproteins, evidenced by lectins blotting, as well as initial tissue requirements dictated the choice of the guanidinium protocol over the other purification protocols. The short purification and the NaPTA protocols failed to produce PrP^Sc^ with adequate purity (Fig S2A, B). The guanidinium protocol was preferred over the SAF preparations, due to its higher yield and significantly lower starting material, time and hardware requirements. Nevertheless, SAF preparations could produce optimal results and ideally when there is no tissue limitation, SAF preparations should be preferable. Interestingly, in preliminary experiments with SAF preparations from human vCJD, bovine BSE, human sCJD and ovine scrapie samples, the lectins recognized only –or mostly- diglycosylated PrP^Sc^, as opposed to human sCJD and ovine scrapie, in which both the di- and the monoglycosylated forms were recognized (Fig S1 and data not shown).

Our experimental setup permits the analysis of the glycosylation pattern of PK resistant PrP, which amounts to a large proportion (at least 45–65%) of total PrP^Sc^ [39]. To our knowledge, glycosylation remains unaltered throughout the conversion of PrP^C^ to PrP^Sc^; therefore the observed differences in PrP^Sc^ glycosylation between the various TSEs could be linked to differences in glycosylation of PrP^C^ following TSE infection, or to the preferential conversion of specific subpopulations of PrP^C^ to PrP^Sc^.

Perturbation of the glycosylation machinery in TSE affected cells, which could affect the glycosylation of PrP and of other proteins, has already been proposed [12], [40], [41]. DNA array analysis in GT1 cells indicates a change in the expression pattern of genes governing the synthesis of glycosaminoglycans [42], however the majority of data from DNA arrays in murine scrapie models advocate against major modification of the expression pattern of glycosylation related genes [43]–[45]. A recent study focused on the expression pattern of glycosylation-related genes in the brains and spleens of Tg338 mice, intracerebrally challenged with a mouse adapted scrapie strain [46]. At terminal stage, eight genes were overexpressed in the brain and five were differentially expressed in the spleen. Interestingly, the biological effects linked to the reported overexpression of two genes (*St6gal1* and *Mgat3*) do not match the observed differences in prion protein glycosylation [12]. The authors argue that these genes are not necessarily upregulated in all cells and that the observed expression alterations may not be involved in the disease pathogenesis, but could reflect disease-associated processes, such as inflammation [46]. Taking into consideration these data, it cannot be ruled out that the observed differences in PrP^Sc^ glycosylation are the result of preferential conversion of select PrP^C^ glycoforms to PrP^Sc^.

Despite N-linked glycosylation is not evidently required for the conversion of PrP^C^ to PrP^Sc^ [22], [23] mounting evidence suggests that sugar chains may be important in the modulation of the conversion, affecting the propagation between different species as well as the appearance of strains. Although the primary structure of the protein is a key determinant for the conversion of PrP^C^ to PrP^Sc^ [47], [48], glycosylation may also play a secondary role, as shown in both *in vitro* and *in vivo* experiments [26]–[28]. PrP^Sc^ itself can dictate the formation of strain-specific PrP^Sc^ glycoforms, even if unglycosylated [24], however the final PrP^Sc^ glycopattern can be influenced by the cell and significantly altered by changes in the glycosylation state of PrP^C^ [29]. In agreement with this, a recent study indicated that in CJD PrP^Sc^ glycoforms ratio significantly correlated with the genotype at codon 129 of the prion protein gene [49] Thus, strain-specific PrP^Sc^ glycosylation profiles could arise from a complex interaction between PrP^C^, PrP^Sc^ and the cell, and may indicate the cellular compartment in which the strain-specific formation of PrP^Sc^ occurs [29]. More recent data advocate on the importance of PrP^C^ glycoforms in *in vitro* conversion systems and argue that interactions between different PrP^C^ glycoforms appear to control the efficiency of prion formation in a species-specific manner [26]. In another *in vivo* study, the importance of host PrP^C^ glycosylation was utterly exalted by the findings that transgenic mice with restricted N-linked glycosylation display striking differences in susceptibility with different prion strains, including complete resistance in some occasions [28].

Our results also point towards the occurrence of a complex interaction between PrP^Sc^, host PrP^C^ and tissue compartment. We observed that in both ovine and murine BSE PrP^Sc^, the total monoglycosylated PrP^Sc^ population is enriched in RCA I rather than DSL reacting subpopulations, as opposed to bovine BSE PrP^Sc^ populations, in which DSL and RCA I reacting monoglycosylated PrP^Sc^ subpopulations were equally represented (Fig S5). If the species barrier and the strain phenomena are indeed the result of the effect of different PrP^C^ conformational requirements by PrP^Sc^ [47], then these findings could be an indication that upon propagation of bovine BSE to new hosts, *i.e.* mice and sheep, the strain adapts and ‘selects’ a different portfolio of PrP^C^ molecules, with a different glycan profile and possibly a different structure which facilitates propagation in the new hosts. Taking under consideration PrP^C^ glycosylation differences in the various brain regions, as well as at the cell level, the already proposed link between prion strain neurotropism and PrP^C^ glycosylation [19], [50] appears appealing, despite already having been debated [51]. It is important, however, to point out that most studies on the influence of PrP^C^ and PrP^Sc^ on TSE transmission rely on quantitative data on the relative abundance of the di-, mono- and unglycosylated forms of the protein, without taking under consideration the nature and abundance of the N-linked glycans. We believe that sequencing of PrP glycans purified from different brain areas in combination with lesion profiling studies could add insight into the phenomenon.

PrP^Sc^ purification following the guanidinium protocol, combined with western blotting and lectin staining proved to be a fast and efficient method for quick estimation of glycans N-linked to PrP^Sc^. When this method was applied on tissue samples affected with a variety of TSEs, differences in the relative abundance of the N-linked glycan moieties were found. Although the importance of prion protein glycosylation for TSEs propagation is still elusive and often debated, our findings indicate that possibly specific subpopulations of PrP^Sc^ molecules, characterized by the presence of distinct sugars, facilitate the propagation of each TSE strain. From a practical standpoint, these differences could be useful as additional traits for the discrimination or origin recognition of TSE strains.

## Supporting Information

Figure S1 Blotting of ovine scrapie, bovine BSE and human sCJD SAFs with Sal1, DSL and RCA I. SAFs were prepared from ovine scrapie (Lanes 1, 4, 7), bovine BSE (Lanes 2, 5, 8) and sCJD (Lanes 3, 6, 9) samples and probed with a polyclonal anti-PrP antibody (Sal1, A), DSL (B) and RCA I (C), as described in ‘Materials and Methods’. Equal amounts of starting material were loaded in all lanes and equal exposure times were used for all three panels. Squares (▪): diglycosylated PrP^Sc^; bullets (•): monoglycosylated; arrow (→): 25 kDa molecular mass marker.
(0.66 MB TIF)

Figure S2 Comparison of three PrP^Sc^ purification protocols. A normal (Lanes 1, 3, 5) and an ovine scrapie sample (Lanes 2, 4, 6) were treated with the NaPTA (A), short (B) or guanidinium protocols (C) and then electrophoresed, electrotransferred and blotted with 6H4, DSL and RCA I, as described in ‘Materials and Methods’. (D) densitometric analysis of the PrP^Sc^ glycoforms following 6H4 staining. Each point represents the percentage of immunoreactivity of the di- and monoglycosylated forms of the protein, versus total immunoreactivity (di-, mono- and unglycosylated forms). Despite all three purification protocols provide PrP^Sc^ with comparable purity and glycoform ratios, only the guanidinium protocol is compatible with lectin staining, as evidenced by the interfering glycoproteins recognized by the lectins following either the NaPTA or the short purification protocol. Equal amounts of starting material were loaded in all lanes for each purification protocol. Solid squares (▪): diglycosylated PrP^Sc^; bullets(•): monoglycosylated; arrowheads(▸): interfering glycoproteins; arrow(→): 25 kDa molecular mass marker; open square(□): NaPTA purified PrP^Sc^; open diamond(◊): short protocol purified PrP^Sc^; solid diamond(⧫): Guanidinium purified PrP^Sc^.
(1.79 MB TIF)

Figure S3 Small differences in the apparent molecular mass of the monoglycosylated PrP^Sc^ band emerge following 6H4 and DSL blotting. PrP^Sc^ was purified with the guanidinium protocol from two representative ovine scrapie samples and then electrophoresed, electrotransferred and probed with 6H4 (A) or DSL (B) as described in ‘Materials and Methods’. The DSL-probed, monoglycosylated PrP^Sc^ band is sharper and corresponds to a portion only of the 6H4 probed monoglycosylated PrP^Sc^ band, with higher apparent molecular mass. This difference in the apparent molecular masses should be attributed to the binding of the lectin on a subpopulation of the PrP^Sc^ molecules presenting the ‘suitable’ sugar moiety for recognition. On the contrary, the antibody binds all the PrP^Sc^ molecules present. Equal amounts of starting material were loaded on all lanes. Squares: diglycosylated PrP^Sc^; bullets (•): monoglycosylated; arrow(→): 25 kDa molecular mass marker, left bracket ({): size range of monoglycosylated PrP^Sc^ after 6H4 blotting; right bracket(}): size range after DSL blotting.
(0.46 MB TIF)

Figure S4 Atypical (Nor98) scrapie. P4 (A, C), DSL (B) and RCA I (D) blotting of PrP^Sc^ purified from the cortex of classical (Lane 1) and Nor98 scrapie samples (Lanes 2–5) with the guanidinium protocol. Equal amounts of starting material were loaded in all lanes. Each membrane was first probed with one lectin (DSL, panel B; RCA I, panel D) and then with P4 (panels A, C respectively), as described in ‘Materials and Methods’. The different pattern in classical scrapie samples in this figure, compared to the one in the other figures, should be attributed to the different part of the brain used. Solid squares (▪): diglycosylated PrP^Sc^ (classical scrapie); bullets (•): monoglycosylated (classical scrapie); open diamonds (◊): PrP^Sc^ associated bands (Nor 98 samples); solid diamonds (⧫): putative PrP^Sc^ associated bands (Nor 98 samples); open squares (□): putative lectin-reacting, C-terminal PrP^Sc^ fragments; arrows (→): 25, 16.5 and 6.5 kDa molecular mass markers. The remaining, non marked lectins-reactive bands, most probably are interfering glycoproteins.
(2.33 MB TIF)

Figure S5 Glycosylation differences among bovine, ovine and murine BSE. The abundance of RCA I and DSL reacting PrP^Sc^ subpopulations in bovine BSE (BSE), ovine BSE (oBSE) and murine BSE (mBSE), guanidinium protocol-purified PrP^Sc^ was estimated by computing the IOD_RCA I_/IOD_DSL_ [integrated optical density of the RCA I reactive band (IOD_RCA I_)/integrated optical density of the DSL reactive band (IOD_DSL_)] ratio for each of the di- and monoglycosylated PrP^Sc^ bands, as well as for the total (di- +monoglycosylated PrP^Sc^ bands). PrP^Sc^ populations in ovine and murine BSE appear to be enriched in RCA I reactive subpopulations compared to bovine BSE. This difference is particularly evident in monoglycosylated PrP^Sc^. Columns represent the mean IOD_RCA I_/IOD_DSL_ and error bars the SEM from three individuals.
(1.00 MB TIF)

Table S1 Lectins used in the study. All the lectins were diluted to 10 µg/ml, except for DSL (6 µg/ml) and checked for PrP^Sc^ recognition on SAFs or guanidinium preparations, as outlined in ‘Materials and Methods’. (-) indicates lack of PrP^Sc^ recognition, as assessed by the apparent molecular masses of the bands. Abbreviations used: mBSE: murine BSE; Sc: ovine scrapie; sCJD: human sporadic CJD; vCJD: human variant CJD; BSE: bovine BSE; mSc: murine scrapie; oBSE: ovine BSE; Nor98: atypical Nor98 ovine scrapie.
(0.05 MB DOC)
