# Supplementary figures and images for: Species and Strain Glycosylation Patterns of PrPSc
Source: PLoS One. 2009 May 20;4(5):e5633. doi: 10.1371/journal.pone.0005633 (PMC2680983; doi:10.1371/journal.pone.0005633)

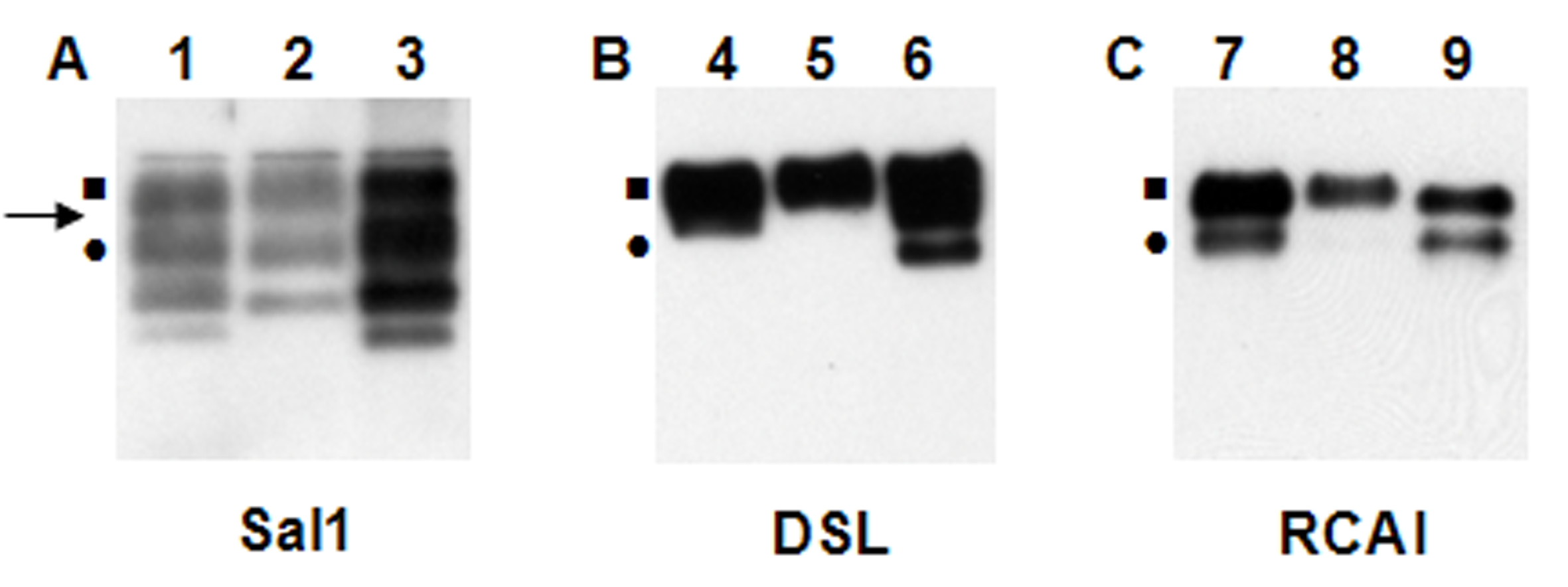

Supplement: Figure S1 — Blotting of ovine scrapie, bovine BSE and human sCJD SAFs with Sal1, DSL and RCA I. SAFs were prepared from ovine scrapie (Lanes 1, 4, 7), bovine BSE (Lanes 2, 5, 8) and sCJD (Lanes 3, 6, 9) samples and probed with a polyclonal anti-PrP antibody (Sal1, A), DSL (B) and RCA I (C), as described in ‘Materials and Methods’. Equal amounts of starting material were loaded in all lanes and equal exposure times were used for all three panels. Squares (▪): diglycosylated PrPSc; bullets (•): monoglycosylated; arrow (→): 25 kDa molecular mass marker. (0.66 MB TIF) [file pone.0005633.s001.tif]

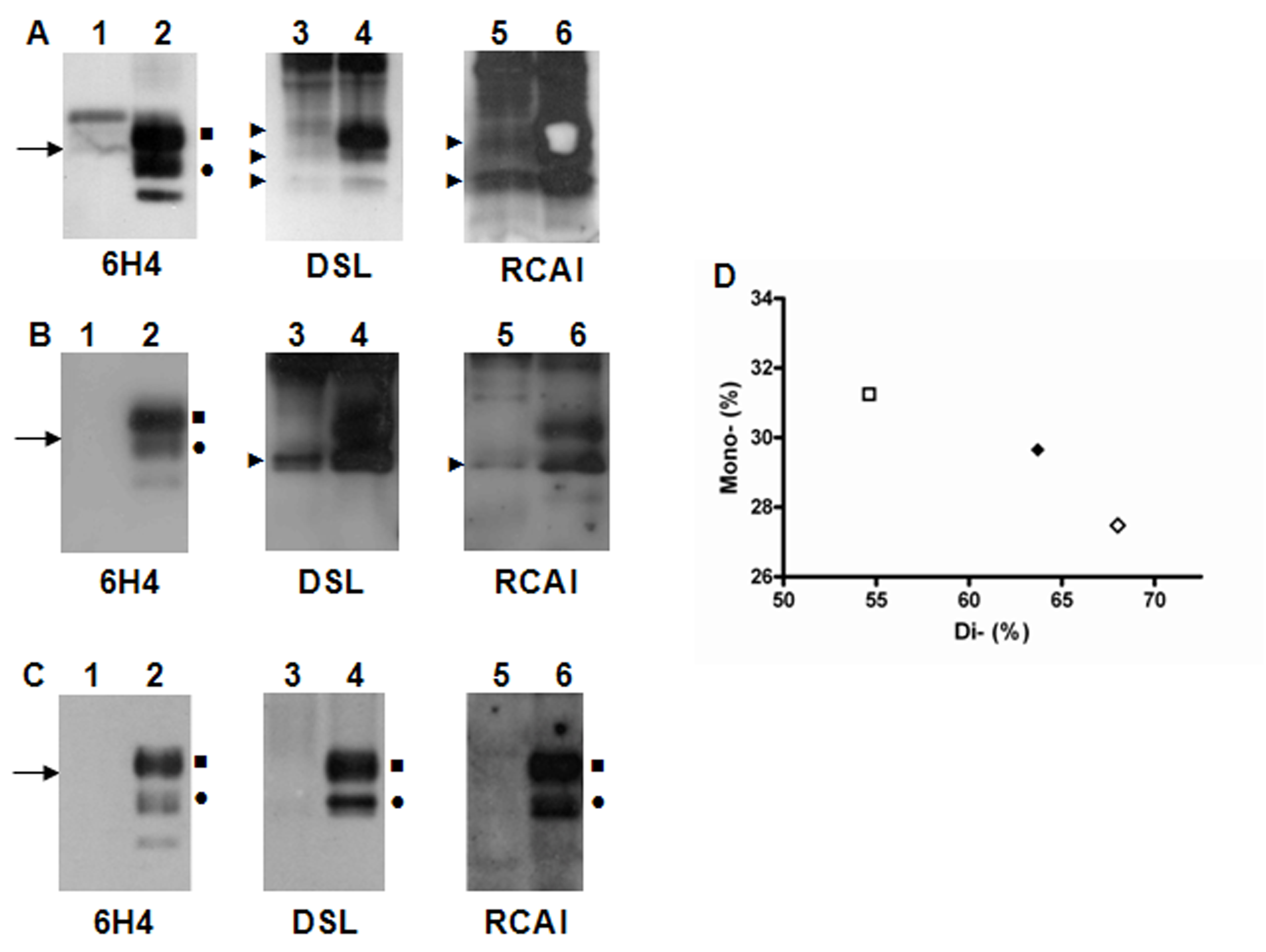

Supplement: Figure S2 — Comparison of three PrPSc purification protocols. A normal (Lanes 1, 3, 5) and an ovine scrapie sample (Lanes 2, 4, 6) were treated with the NaPTA (A), short (B) or guanidinium protocols (C) and then electrophoresed, electrotransferred and blotted with 6H4, DSL and RCA I, as described in ‘Materials and Methods’. (D) densitometric analysis of the PrPSc glycoforms following 6H4 staining. Each point represents the percentage of immunoreactivity of the di- and monoglycosylated forms of the protein, versus total immunoreactivity (di-, mono- and unglycosylated forms). Despite all three purification protocols provide PrPSc with comparable purity and glycoform ratios, only the guanidinium protocol is compatible with lectin staining, as evidenced by the interfering glycoproteins recognized by the lectins following either the NaPTA or the short purification protocol. Equal amounts of starting material were loaded in all lanes for each purification protocol. Solid squares (▪): diglycosylated PrPSc; bullets(•): monoglycosylated; arrowheads(▸): interfering glycoproteins; arrow(→): 25 kDa molecular mass marker; open square(□): NaPTA purified PrPSc; open diamond(◊): short protocol purified PrPSc; solid diamond(⧫): Guanidinium purified PrPSc. (1.79 MB TIF) [file pone.0005633.s002.tif]

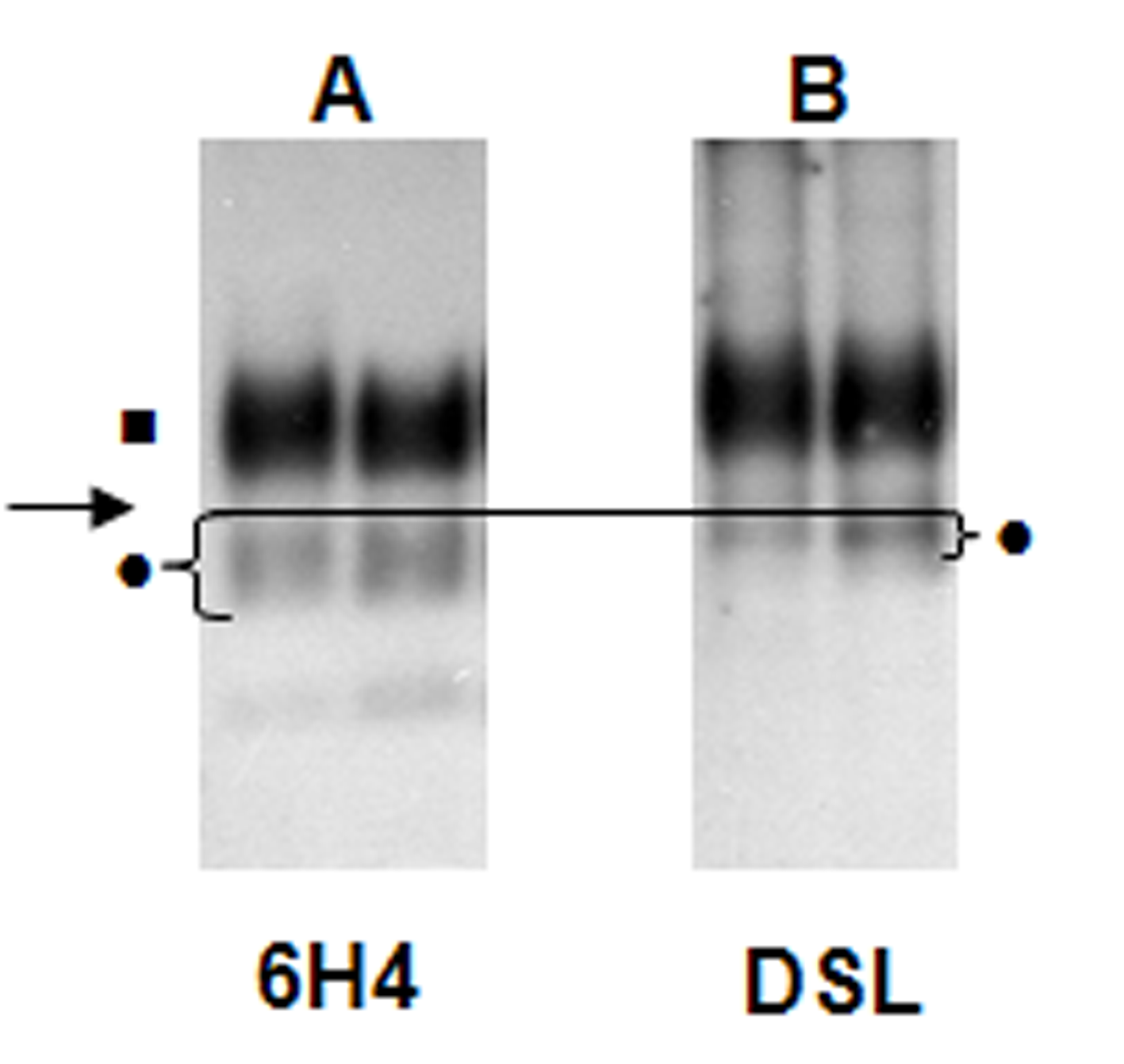

Supplement: Figure S3 — Small differences in the apparent molecular mass of the monoglycosylated PrPSc band emerge following 6H4 and DSL blotting. PrPSc was purified with the guanidinium protocol from two representative ovine scrapie samples and then electrophoresed, electrotransferred and probed with 6H4 (A) or DSL (B) as described in ‘Materials and Methods’. The DSL-probed, monoglycosylated PrPSc band is sharper and corresponds to a portion only of the 6H4 probed monoglycosylated PrPSc band, with higher apparent molecular mass. This difference in the apparent molecular masses should be attributed to the binding of the lectin on a subpopulation of the PrPSc molecules presenting the ‘suitable’ sugar moiety for recognition. On the contrary, the antibody binds all the PrPSc molecules present. Equal amounts of starting material were loaded on all lanes. Squares: diglycosylated PrPSc; bullets (•): monoglycosylated; arrow(→): 25 kDa molecular mass marker, left bracket ({): size range of monoglycosylated PrPSc after 6H4 blotting; right bracket(}): size range after DSL blotting. (0.46 MB TIF) [file pone.0005633.s003.tif]

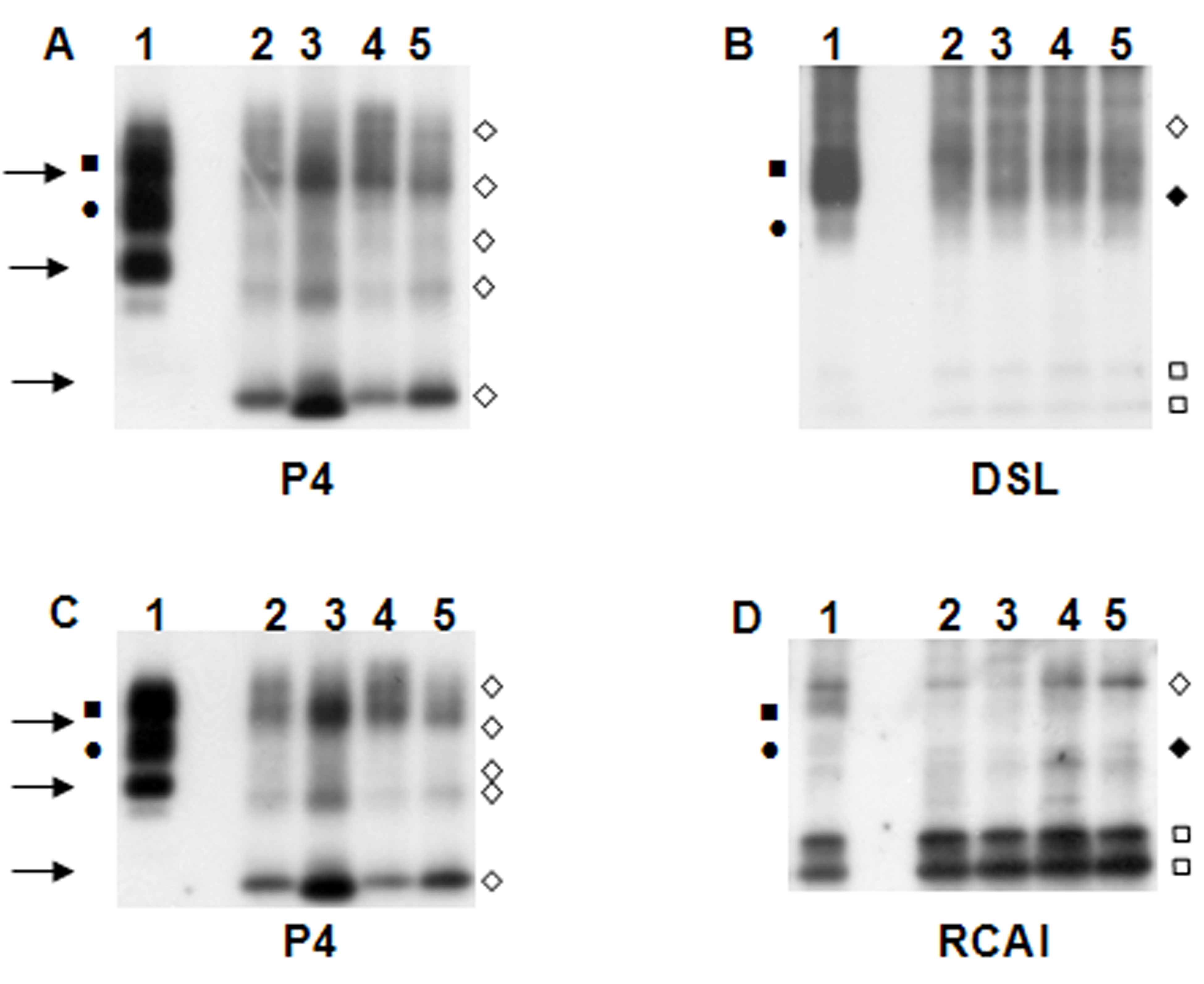

Supplement: Figure S4 — Atypical (Nor98) scrapie. P4 (A, C), DSL (B) and RCA I (D) blotting of PrPSc purified from the cortex of classical (Lane 1) and Nor98 scrapie samples (Lanes 2–5) with the guanidinium protocol. Equal amounts of starting material were loaded in all lanes. Each membrane was first probed with one lectin (DSL, panel B; RCA I, panel D) and then with P4 (panels A, C respectively), as described in ‘Materials and Methods’. The different pattern in classical scrapie samples in this figure, compared to the one in the other figures, should be attributed to the different part of the brain used. Solid squares (▪): diglycosylated PrPSc (classical scrapie); bullets (•): monoglycosylated (classical scrapie); open diamonds (◊): PrPSc associated bands (Nor 98 samples); solid diamonds (⧫): putative PrPSc associated bands (Nor 98 samples); open squares (□): putative lectin-reacting, C-terminal PrPSc fragments; arrows (→): 25, 16.5 and 6.5 kDa molecular mass markers. The remaining, non marked lectins-reactive bands, most probably are interfering glycoproteins. (2.33 MB TIF) [file pone.0005633.s004.tif]

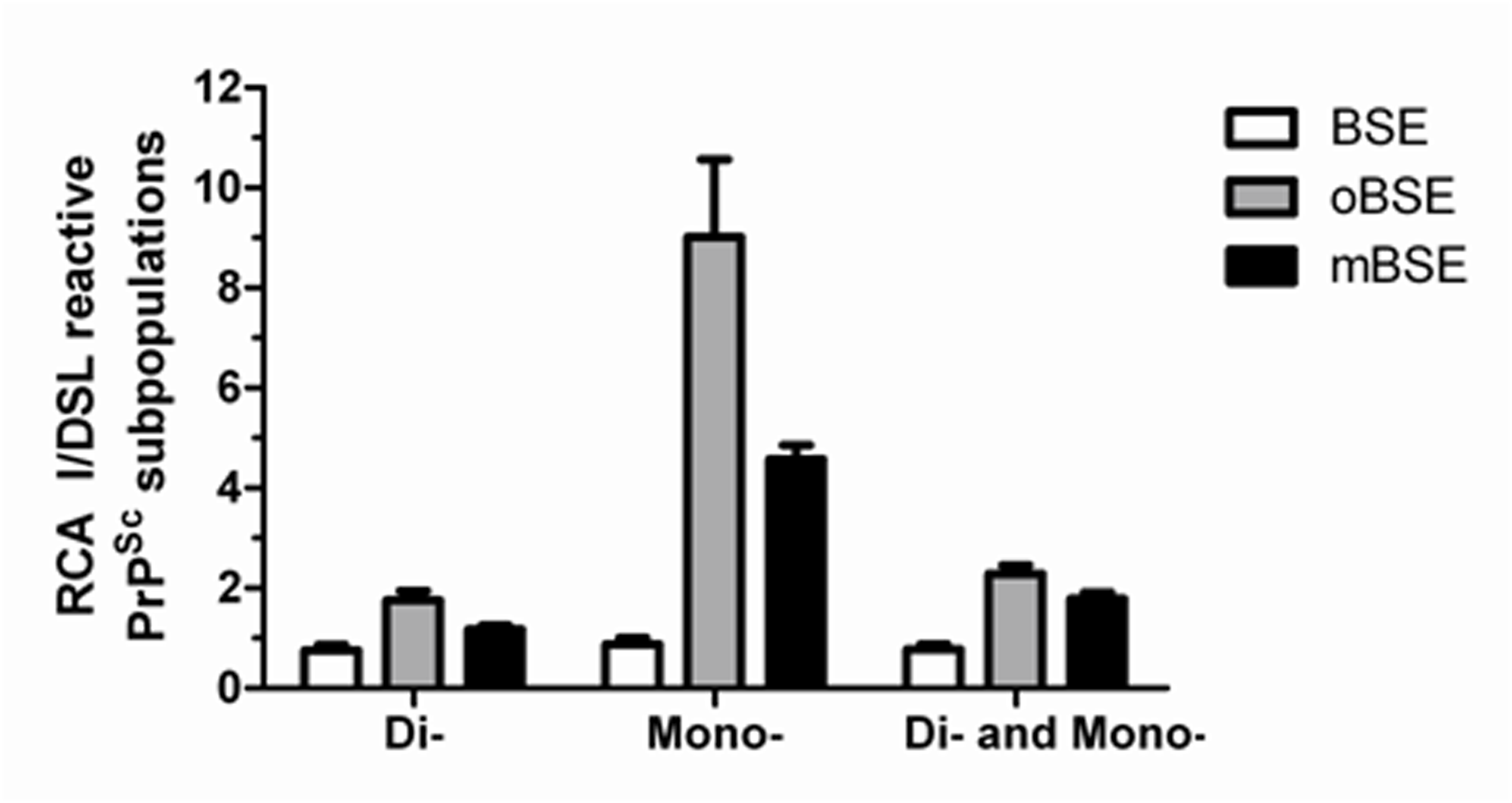

Supplement: Figure S5 — Glycosylation differences among bovine, ovine and murine BSE. The abundance of RCA I and DSL reacting PrPSc subpopulations in bovine BSE (BSE), ovine BSE (oBSE) and murine BSE (mBSE), guanidinium protocol-purified PrPSc was estimated by computing the IODRCA I/IODDSL [integrated optical density of the RCA I reactive band (IODRCA I)/integrated optical density of the DSL reactive band (IODDSL)] ratio for each of the di- and monoglycosylated PrPSc bands, as well as for the total (di- +monoglycosylated PrPSc bands). PrPSc populations in ovine and murine BSE appear to be enriched in RCA I reactive subpopulations compared to bovine BSE. This difference is particularly evident in monoglycosylated PrPSc. Columns represent the mean IODRCA I/IODDSL and error bars the SEM from three individuals. (1.00 MB TIF) [file pone.0005633.s005.tif]
