# Supplementary material for: Species and Strain Glycosylation Patterns of PrPSc
Source: PLoS One. 2009 May 20;4(5):e5633. doi: 10.1371/journal.pone.0005633 (PMC2680983; doi:10.1371/journal.pone.0005633)
Supplement: Table S1 — Lectins used in the study. All the lectins were diluted to 10 µg/ml, except for DSL (6 µg/ml) and checked for PrPSc recognition on SAFs or guanidinium preparations, as outlined in ‘Materials and Methods’. (-) indicates lack of PrPSc recognition, as assessed by the apparent molecular masses of the bands. Abbreviations used: mBSE: murine BSE; Sc: ovine scrapie; sCJD: human sporadic CJD; vCJD: human variant CJD; BSE: bovine BSE; mSc: murine scrapie; oBSE: ovine BSE; Nor98: atypical Nor98 ovine scrapie. (0.05 MB DOC) [file pone.0005633.s006.doc]

| Lectin | Specificity | Tested on | PrP^Sc^ Reactivity |
| --- | --- | --- | --- |
| Concanavalin A | α-linked mannose | mBSE, Sc, sCJD, BSE SAFs | - |
| Datura stramonium agglutinin | β→1,4 linked N-acetylglucosamine oligomers | mBSE, Sc, sCJD, vCJD, BSE SAFs | di- and mono- glycosylated PrP^Sc^ (mostly the di- in BSE and vCJD) |
|  |  | mBSE, mSc, Sc, sCJD, BSE, oBSE and Nor98 guanidinium preps | di- and mono- glycosylated PrP^Sc^ in mBSE, mSc, Sc, sCJD, BSE, oBSE samples; multi-band pattern in Nor98 guanidinium preps |
| Erythrina cristagalli lectin | galactosyl (β→1,4) N-acetylglucosamine | Nor98 guanidinium preps | - |
| Griffonia simplicifolia lectin I | α-N-acetylgalactosamine residues and α-galactose residues | Nor98 guanidinium preps | - |
| Jacalin | o-linked oligosaccharides; preference for galactosyl (β→1,3) N-acetylgalactosamine | mBSE, Sc, sCJD, BSE SAFs | - |
| Lens culinaris agglutinin | α-linked mannose residues | mBSE, Sc, sCJD, BSE SAFs | weak recognition of the di- and mono- glycosylated bands |
|  |  | Nor98 guanidinium preps | - |
| Lycopersicum esculentum lectin | N-acetylglucosamine oligomers; preference for trimers and tetramers | mBSE, Sc, sCJD, BSE SAFs | - |
|  |  | Nor98 guanidinium preps | - |
| Maackia amurensis lectin II | sialic acid in α→2,3 linkage | mBSE, Sc, sCJD, BSE SAFs | - |
|  |  | Nor98 guanidinium preps | - |
| Pisum sativum agglutinin | α-linked mannose-containing oligosaccharides; preference for those with an N-acetylchitobiose-linked α-fucose residue included in the receptor sequence | Nor98 guanidinium preps | - |
| Ricinus communis agglutinin I | oligosaccharides ending in galactose and N-acetyl-galactosamine; may also interact with N-acetylgalactosamine | mBSE, Sc, sCJD, vCJD, BSE SAFs | di- and mono- glycosylated PrP^Sc^ (mostly the di- in BSE and vCJD) |
|  |  | mBSE, mSc, Sc, sCJD, BSE, oBSE and Nor98 guanidinium preps | di- and mono- glycosylated PrP^Sc^ in mBSE, mSc, Sc, sCJD, BSE, oBSE samples; multi-band pattern in Nor98 guanidinium preps |
| Sambucus nigra agglutinin | sialic acid attached to terminal galactose in α→2,6, and to a lesser degree α→2,3, linkage | mBSE, Sc, sCJD, BSE SAFs | di- and mono- glycosylated PrP^Sc^, but other interfering bands with similar molecular weights were also recognized |
| Solanum tuberosum lectin | oligomers of N-acetylglucosamine | mBSE, Sc, sCJD, BSE SAFs | - |
|  |  | Nor98 guanidinium preps | - |
| Ulex europaeus agglutinin I | glycoproteins and glycolipids containing α-linked fucose residues | mBSE, Sc, sCJD, BSE SAFs | - |
| Vicia villosa lectin | α- or β-linked terminal N-acetylgalactosamine; preference for the Tn antigen | Nor98 guanidinium preps | - |
| Wheat germ agglutinin | N-acetylglucosamine; preference for dimers and trimers | mBSE, Sc, sCJD, BSE SAFs | faint staining of the diglycosylated PrP^Sc^ band |

**Table S1: Lectins used in the study.** All the lectins were diluted to 10 μg/ml, except for DSL (6 μg/ml), and checked for PrP^Sc^ recognition on SAFs or guanidinium preparations, as outlined in 'Materials and Methods'. (–) indicates lack of PrP^Sc^ recognition, as assessed by the apparent molecular masses of the bands. Abbreviations used: mBSE: murine BSE; Sc: ovine scrapie; sCJD: human sporadic CJD; vCJD: human variant CJD; BSE: bovine BSE; mSc: murine scrapie; oBSE: ovine BSE; Nor98: atypical Nor98 ovine scrapie.
